# Silicon Substrate Strained and Structured via Cavitation Effect for Photovoltaic and Biomedical Application

**DOI:** 10.1186/s11671-016-1400-2

**Published:** 2016-04-11

**Authors:** Rada K. Savkina, Aleksandr I. Gudymenko, Vasyl P. Kladko, Andrii A. Korchovyi, Andrii S. Nikolenko, Aleksey B. Smirnov, Tatyana R. Stara, Viktor V. Strelchuk

**Affiliations:** V. Lashkaryov Institute of Semiconductor Physics, National Academy of Sciences of Ukraine, Prospect Nauky, 41, Kyiv, 03028 Ukraine

**Keywords:** Micro- and nanoscale pattern formation, Cavitation, Silicon substrate, Photovoltaic effect, Biocompatibility, 47.55.dp, 81.16.Rf

## Abstract

A hybrid structure, which integrates the nanostructured silicon with a bio-active silicate, is fabricated using the method of MHz sonication in the cryogenic environment. Optical, atomic force, and scanning electron microscopy techniques as well as energy dispersive X-ray spectroscopy were used for the investigation of the morphology and chemical compound of the structured surface. Micro-Raman as well as X-ray diffraction, ellipsometry, and photovoltage spectroscopy was used for the obtained structures characterization. Ellipsometer measurements demonstrated the formation of the layer with the thicknesses ~700 nm and optical parameters closed to SiO_2_ compound with an additional top layer of the thicknesses ~15 nm and the refractive index ~1. Micro-Raman investigation detects an appearance of Ca–O local vibrational mode, and the stretching vibration of SiO_4_ chains characterized the wollastonite form of CaSiO_3_. A significant rise in the value and an expansion of the spectral range of the surface photovoltage for silicon structured via the megasonic processing was found. The concept of biocompatible photovoltaic cell on the base of Si\CaSiO_3_ structure for the application in bioelectronics was proposed.

## Background

Successful manipulation of the material properties on the small-scale level has been driven by the development of new methods that provide control over the size and morphology of nano/microstructure. One of such modern methods is based on the application of the acoustic cavitation. Acoustic cavitation is a phenomenon observed at the ultrasound transmission through a liquid, causing gas bubbles to oscillate, grow, and violently implode. This is giving rise to extreme, but localized, conditions within the collapsed cavities [1]. Creation of the extreme conditions inside the collapsing bubble serves as the origin of the majority sonochemical phenomena in the liquid or liquid-solid interface.

The oscillating bubbles (typically tens of micrometers (μm) in radius *R* at kilohertz (kHz) frequency cavitation) accumulate ultrasonic energy and release it during collapse in the form of a temperature of about 5000 K and pressure of several hundreds of megapascals (MPa). At 1 MHz, the active cavitation bubbles are so small that they are difficult to observe in a direct way. At the same time, localization and dynamics of cavitation bubbles induced by focused MHz ultrasound are rather specific but promise to be extremely effective thanks to the energy concentration at the focal. The study of the bubble dynamics in the frame of the Rayleigh-Plesset equation has shown that the mean radius *R* of the cavitating bubble at a MHz frequency cavitation is in the subnanometer range. For example, *R* = 300–600 nm for argon and xenon at *f*_US_ = 1 MHz [2]. And, the minimum radius of the collapsing bubble, caused by the van der Waals hard-core volume, is reduced to the value of ~*R*/9, i.e., is in a nanoscale region. Thus, the collapsing bubble transmits a huge amount of power density to a solid surface at the nanometer scale.

The interaction features between acoustic waves and semiconductors permit to manipulate by the crystal properties for a stable improvement of the existing electronic devices as well as for developing of new ones. But, the behavior of the semiconductor surfaces under the acoustic cavitation remains a little studied. For example, sonication of silicon is principally focused on the cleaning of the surface [3] or on the development of the porous luminescent structures [4]. It also demonstrated the effectiveness of the back-side damage gettering in silicon introduced by a cavitating jet [5].

In our previous work, the suitable condition of the cavitation processing for causing the incorporation of the nitrogen atoms into the GaAs lattice and the formation of an ordered system of (GaN)m(GaAs)n clusters (*m* = *n* = 1) have been successfully established [6, 7]. It was also revealed that the characteristic dimension of the peculiarities on the semiconductor surface depended on the exposure parameters and can be controlled (from micron- to nanoscale dimension) by the regulation of the acoustic frequency. In this investigation, we focus on an attempt to drive the chemical and structural transformations on the silicon surface by the ultrasonic cavitation effect. Our choice is dictated by the fact that structured (in particular, porous) silicon is an ideal material for the design of biologically interfaced devices [8–10]. In our paper, we propose the concept of a new biocompatible photovoltaic (PV) cell on the base of silicon substrate which was strained and structured via cavitation effect.

## Methods

Materials used in this study were boron-doped (100)-oriented *p*-type silicon wafers of diameter about 76.2 mm grown by the liquid-encapsulated Czochralski method. Samples were cut into 5 × 5 mm squares and were cleaned for 10 min in ethanol and then in ddH_2_O (water for analytical laboratory use, ISO 3696:1987). The initial surface was found to be totally flat, devoid of defects, and with a measured roughness lower than 1 nm. The roughness was determined by atomic force microscopy (AFM) on a few randomly chosen areas of 40 × 40 μm^2^. X-ray diffraction (XRD) pattern studies for the initial samples denoting the existence of a small amount of amorphous phase on the Si substrate surface were investigated.

The Si samples to study were sorted into two groups. The first group was treated by the cavitation impact in cryogenic liquid such as nitrogen (LN_2_). The second group of silicon samples was sonicated in LN_2_ with an addition to the reactor vessel of a considerable amount of calcium. After sonication, both groups were annealed in the nitrogen vapor at 1100 °C for 2 h.

For cavitation activation, we used a high-frequency system (MHz) with focused energy resonator described elsewhere [6]. The experimental setup consisted of a reactor vessel and US equipment. Semiconductor target was placed inside the acoustically driven cell in the focus region. A ceramic piezoelectric transducer (PZT-19) with a resonant frequency of 3 MHz (or 6 MHz) acoustically drove the cell. The output voltage of the US generator did not exceed 5 V, and the initial value of the acoustic intensity *W*_US_ did not exceed 1 W/cm^2^. The intensity gain of the acoustic system was about 58. The maximal value of the pressure was about ~8 bars at the focus of the acoustic system.

All processed surfaces were examined after fixed cavitation intervals using optical and atomic force microscopy (Digital Instruments NanoScope IIIa operating in the tapping mode). The surface morphology was examined using scanning electron microscope JSM-6490, Jeol (SEM) supplemented by energy dispersive X-ray analysis (EDAX) (EDS JED 2300 detector) for the chemical analysis. All samples in the initial state and after the cavitation treatment and annealing were characterized by measuring their surface photovoltage (SPV) spectra. The optical characteristics of the typical annealed sample were studied by ellipsometry. The measurements were performed on a laser (*λ* = 632.8 nm) photoelectric compensation null ellipsometer (LEF 3G-1). The ellipsometric parameters *∆* and *ψ* were determined from the results of multi-angle measurements in a range of incidence angle *ϕ* = 50°–75°. The structural characterization of the silicon samples was carried out by XRD in the standard symmetric reflection geometry using CuK_α_ radiation. X-ray rocking curves and symmetric ω-2θ scan for samples investigated before and after sonication were obtained using a PANalytical X’Pert PRO triple-axis X-ray diffractometer. The CuK_α1_ radiation with a wavelength of 0.15418 nm was separated out using a four-bounce (440) Ge monochromator. Micro-Raman spectra in the 100- to 2000-cm^−1^ spectral range at room temperature were registered in backscattering geometry using triple Raman spectrometer T-64000 HORIBA Jobin Yvon, equipped with cooled charge-coupled device detector. The line 488 nm of Ar-Kr ion laser was used for excitation. Exciting radiation with power of 1–2 mW was focused on the sample surface to the spot of 1 μm in diameter.

## Results and Discussion

Our preliminary study of a MHz frequency sonication of the silicon target has shown changes in the surface morphology and chemical composition as well as improvement of the photoelectric properties of the silicon samples exposed to the cavitation processing [11, 12]. Really, in contrast to the kHz frequency processing mode [13] resulted in the erosion on the silicon surface after a long-time treatment, the MHz frequency sonication does not lead to the semiconductor surface degradation, cracks formation, and fracture. Moreover, we observed the formation of the micron- and nanoscale complex structures after the first 10–15 min of the testing time. Their characteristic dimension is decreased at a frequency rise that can be explained by the decrease of the mean size of a cavitating bubble [14].

**Fig. 1:**
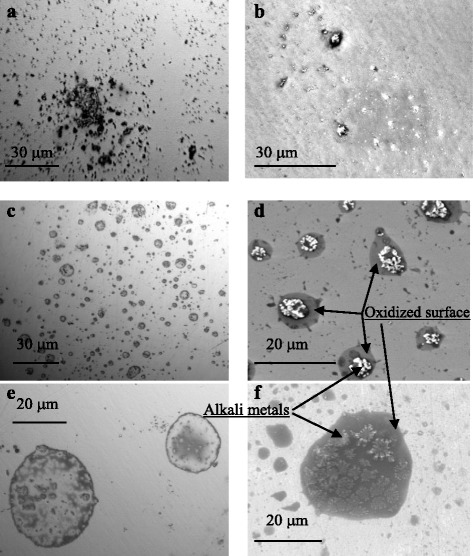
Optical and SEM (7 kV, ×2000) images of Si samples (**a–f**). Samples from the first group were sonicated in the liquid nitrogen at 6 MHz. Weight percent of alkali metals determined by EDAX technique inside the structured regions of silicon samples is 0.6–1.37 % for Na, 0.16–2.5 % for K, and 0.19–2.07 % for Ca. Weight percent of the oxygen in some areas reached 12 %

Figure 1a–f shows the typical optical and SEM images of silicon samples from the first group after MHz frequency sonication. All samples exhibited negligible surface modification up to at least the first 5 min of the MHz sonication. After about 10 min of the testing time, the optical (Fig. 1a) and electron (Fig. 1b) microscopy reveals small pits on the surface. After 15–30 min of sonication, the character of surface modification becomes more complex. Together with small pits, rings about 5–30 μm in diameter are formed (Fig. 1c–f). The microscopy investigations revealed the growth of dendrite-like objects inside the structured region of Si samples during sonication.

EDAX measurements have shown that ultrasonically structured regions have a silicon nature and are surrounded with an oxidated surface (deep gray, Fig. 1d, f). Occurrence of metals such as Na, K, and Ca inside the structured regions was found also. It should be noted that minor peaks corresponding to the elements K and Ca were identified by EDS analyses on GaAs surface after the cavitation exposure [6]. We guess that they could have been dislodged from the walls of the experimental cell as well as from the technical nitrogen directly. In other words, it is a feature of our experimental procedure.

However, we found not only a surprising amount of impurities on the silicon surface but compounds having a crystal structure such as Na_2_Ca_3_(Si_3_O_10_) and CaSiO_3_ [15], the last of which exhibits good bioactivity and biocompatibility. Thus, the fabrication of a hybrid structure, which integrates the structured silicon with a bio-active silicate, is quite realistic under the cavitation processing. In order to make it, the second group of silicon samples was prepared by a MHz frequency processing in LN_2_ with an addition of calcium to the reactor vessel.

AFM topographic measurements allow one to monitor the morphological evolution of the sonicated Si samples and demonstrate that the structured surface occurs significantly below the initial surface level. The relative increase of the roughness of the surface is observed. The corresponding surface roughness was quantified using root mean square (RMS) average of height deviations parameter. An increasing RMS roughness is appearing with the duration of the acoustic cavitation.

**Fig. 2:**
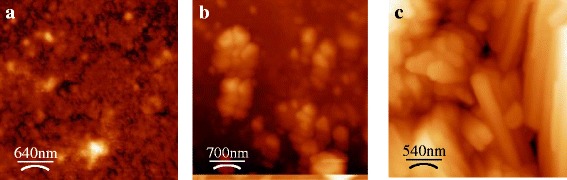
Typical AFM images of the surface evolution during the 6-MHz sonication (15 W/cm^2^) of Si samples from the second group. **a** 10 min treatment in LN_2_, RMS roughness is ~8 nm; **b** 20 min treatment in LN_2_, RMS roughness is ~35 nm; **c** 35 min treatment in LN_2_

The AFM images of a typical sample investigated from the second group is shown in Fig. 2 that demonstrate the surface evolution during a MHz frequency cavitation action and formation of subnanometer objects (with the characteristic size about ∼200 nm) inside structured regions. Prolonged sonication leads to the modification of the object shape and to the rise of their size (see Fig. 2c).

More detailed information about the processed samples were obtained from XRD measurements. The initial XRD pattern reveals only Si (400) diffraction peak that indicates (100) orientation of the silicon substrate. A phase composition of the subsurface layer of the sonicated and annealed Si samples was studied by X-ray diffraction in the grazing geometry. The incidence angle of the X-ray beam was chosen as 5°. For the typical sample after sonication and annealing, a series of peaks corresponding to SiO_2_ lattice reflections of Cu_Kα_ photons was observed. In particular, such polymorphs of SiO_2_ as quartz and cristobalite were found. In addition, peaks corresponding to Ca and calcium compounds (CaSiO_3_ and Ca_2_SiO_4_) were found.

**Fig. 3:**
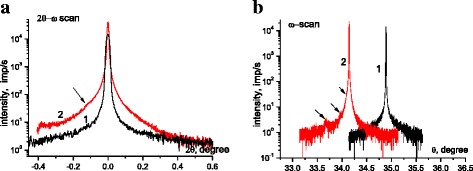
X-ray rocking curves for typical sample after sonication (*1*) and after sonication and annealing (*2*) (**a, b**)

X-ray rocking curves (RC) for silicon samples before and after sonication were obtained from the symmetrical 2θ-ω scanning. The initial RC has a symmetric form with a full width at half maximum (FWHM) *Δ*_FWHM_ ~12″, which is close to the theoretically predicted value of FWHM for a perfect silicon crystal. After cavitation processing, a rocking curve is broadened out (*Δ*_FWHM_ has increased to 64.8″, see Fig. 3a, curve 1) that indicates an appearance of structural defects. After annealing, RC exhibits an asymmetry with features on the left side of the diffraction profile (see Fig. 3a, curve 2, Δ_FWHM_ = 32″). The angular position of this feature is *Δθ* = 421″, which corresponds to the maximum strain that prevails in the sample, having a value of *ε* = *Δθ*cos*θ* = 10^−3^ (*σ*~0.18 GPa). Moreover, the appearance of asymmetry and features in the *q*_x_ direction (ω-scan) point out to the formation of misorientation-type defects as a result of the post-sonication annealing (see Fig. 3b, curves 1 and 2).

The study of the optical characteristics of the samples, carried out by ellipsometry, points to the formation of the layer with the thicknesses ~700 nm and optical parameters closed to SiO_2_ compound (refractive index *n*~1.46 and extinction coefficient *k*→0 at *λ* = 632.8 nm) after sonication and thermal annealing in the nitrogen vapor. The accurate interpretation of the ellipsometer results became possible only after introducing an additional top layer with the thicknesses ~15 nm and the refractive index ~1.

**Fig. 4:**
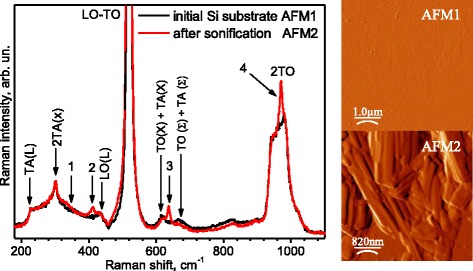
Micro*-*Raman spectra measured around localized defects after MHz sonication (15 W/cm^2^, 30 min) and annealing: *1* 336 cm^−1^, *2* 412 cm^−1^, *3* 642 cm^−1^, *4* 971 cm^−1^. Spectrum of the untreated silicon is depicted as *black*. On the right of the micro-Raman spectra, the AFM images of the respective regions are depicted

Micro-Raman investigations of the untreated crystalline silicon samples exhibit a cubic diamond structure (space group Fd-3m) characterized by a one first-order Raman active phonon located at the center Γ point of the Brillouin zone corresponding to a phonon wave vector of ~520 cm^-1^ (phase conventionally labeled Si-I) and measured full width of 3.8 cm^-1^ (see Fig. 4). In addition, the first-order scattering from transfer TA (L) acoustical phonons at 230 cm^−1^ and from optical phonon LO(L) at 430 cm^−1^ as well as the second-order scattering from transfer 2TA acoustical phonons at 300 cm^−1^ [16] are observed. The peak at 620 cm^−1^ probably corresponds to the combination TO(X) + TA(X) modes, and the weaker peak at 670 cm^−1^ corresponds to TO (Σ) + TA (Σ) [17]. Note that the peak at ∼430 cm^−1^ can be attributed to polymorphic metastable phase of crystalline Si−III (bc8, body-centered cubic structure, eight atoms per unit cell) in accord with the literature [18].

After sonication, spectra measured around localized defects were found to present a broadened LO-TO(Γ) c-Si band, with a measured full width at half maximum up to Δ_FWHM_~4.54 cm^-1^ (see Fig. 4). The LO-TO peak broadening can be attributed to both an increase in the density of defects within the crystals and to a phonon confinement effect resulting from the nanocrystalline silicon formation (nc-Si) [17]. Its line shape becomes asymmetric with a little tail on the low-energy side extending to 470–480 cm^−1^ for all spectra which indicates a partial amorphous-like structure.

Discussed features characterize all samples after sonication and annealing. At the same time, a close inspection of the Raman spectra for the second group of samples (see Fig. 4) detects an appearance of Ca–O local vibrational mode at about 336 and 412 cm^−1^, and Raman features at 642 and 971 cm^−1^ corresponded the stretching vibration of the monomer SiO_4_ and the stretching vibration of the chain SiO_4_ tetrahedron, respectively [19]. These modes, as it is known [19], characterize the wollastonite form of CaSiO_3_.

As it is known, flat silicon is reflective even in the visible wavelength range, limiting the sensitivity of silicon photodetectors and the efficiency of solar cells. At the same time, the nanoscale architectures play a very important role in enhancing device efficiency. For example, structuring silicon with the femtosecond laser pulses contributes several advantages to current silicon solar cells most likely due to the decreased reflectance because of the surface texture [20]. Based on the extremely large surface areas of the nanocrystals, combined with their deliberate engineering of the energy-band alignment, it is possible to obtain nanoscale PV devices with excellent performance.

**Fig. 5:**
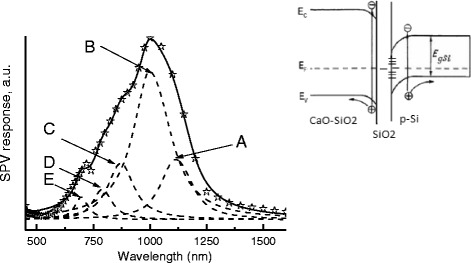
Surface photovoltage spectra of the typical silicon sample exposed to the acoustic cavitation in cryogenic fluid and annealing in the nitrogen vapor. *Dots* indicate the experimental data, and *solid lines* present the result of the fitting procedure. The *thin-dash lines* show approximation by the Lorentzian model. *Inset*: the energy band diagram of the Si/SiO2/(CaO-SiO2) structure in dark conditions

Thus, expecting improvement in the photoelectric properties of silicon samples exposed to the MHz frequency sonication in the LN_2_, we have investigated their spectral distribution of the SPV. These measurements were performed using a lock-in scheme with a modulation at 300 Hz at low level of homogeneous excitation by a monochromatic light in a wavelength range of 0.45–2 μm. A significant rise in the value and an expansion of the spectral range of the photosensitivity occurs. The measured value of the photovoltage after MHz sonication was about 6 mV at the light power density ~0.01 W mm^−2^, whereas the untreated Si samples exhibit very low level of the photovoltage response. SPV spectrum (a broad band of the photosensitivity between 500 and 1300 nm) of the typical sample investigated after MHz sonication and annealing is shown in Fig. 5. The photovoltage spectrum had complicated shape that could be described by a sum of five Lorentzian components. The energy positions of components were 1.1 (peak A), 1.2 (peak B), 1.42 (peak C), 1.58 (peak D), and 1.8 eV (peak E). An abrupt increase of photoresponse at about 1.1 eV corresponding to the silicon band gap *E*_g_ at 300 K (peak A, Fig. 5) was seen. We think that another SPV features (peaks B–E) reflect the excitation process in oxide-based multilayer structure produced on silicon surface after cavitation processing in the LN_2_.

As is known, a surface photovoltage arises whenever light-induced excess charge carriers are separated within the space charge region spontaneously created under a free semiconductor surface or on the interface of the heterojunction. Evidently, the measured SPV signal is related to the surface inhomogeneity caused by the treatment of the semiconductor in the cavitating fluid. On the basis of the ellipsometer and XRD measurements, a complex oxide-based multilayer structure consisting of the Si/SiO_2_ interface with surface silicate (CaO-SiO_2_) has formed as a result of the cavitation processing. Figure 5 shows the energy band diagram of the Si/SiO_2_/(CaO-SiO_2_) structure in dark conditions. The electric field that appears in the region of inhomogeneity at the illumination (in this case, inhomogeneous chemical composition) accelerates the photogenerated charge carriers, which leads to the spatial separation of carriers possessing opposite signs. It should be also noted that a wide-gap feature at 1.8 eV (Fig. 5, peak E) compared to the band gap of amorphous silicon (1.7–1.8 eV band gap).

## Conclusions

We have demonstrated that the acoustic cavitation induced in the liquid nitrogen by the focused ultrasound activates the extremely effective transformation on the semiconductor surface at the nanometer scale. Deformation and structurization of the silicon samples were observed. Based on micro*-*Raman and SPV spectroscopy results, we obtained an improvement in the photoelectric properties and a new chemical phase formation on silicon surface exposed to the MHz frequency sonication in the liquid nitrogen. Thus, a possibility of the fabrication of a hybrid structure Si\CaSiO_3_, which integrates the nanostructured silicon with bio-active silicate, using a simple and low-cost method has been demonstrated. It is necessary to note that one of the most critical issues in realizing the potential of bio-electronic devices is their biocompatibility. New methods for harvesting and storing energy to power mobile and implantable devices are required also. Using the ultrasonic cavitation to manipulate semiconductor surfaces on a small scale allowed us to obtain silicon with a unique combination of PV properties and biocompatible material on the surface. As a result, integration, a key element of semiconductor electronics with biological systems, becomes simpler. This motivates further efforts towards the development of the biocompatible PV cell based on Si\CaSiO_3_ structure for possible application in bioelectronics.

## Abbreviations

AFM: atomic force microscopy
EDAX: energy dispersive X-ray analysis
FWHM: full width at half maximum
LN_2_: liquid nitrogen
PZT: piezoelectric transducer
RMS: root mean square
SEM: scanning electron microscopy
SPV spectra: surface photovoltage spectra
US: ultrasonic
XRD: X-ray diffraction
